# Thiamylal serum concentration for refractory convulsive status epilepticus while associated decreased concentrations of concomitant antiepileptics: a case report

**DOI:** 10.1186/s40780-024-00362-w

**Published:** 2024-07-12

**Authors:** Kazutaka Oda, Tomomi Katanoda, Hitomi Arakaki, Taiki Katsume, Kaho Matsuyama, Hirofumi Jono, Hideyuki Saito

**Affiliations:** 1 Department of Pharmacy, Kumamoto University Hospital, 1-1-1Chuo-Ku, Kumamoto City, HonjoKumamoto, Japan; 2 Department of Clinical Pharmaceutical Sciences, Graduate School of Pharmaceutical Sciences, Kumamoto University, 1-1-1Chuo-Ku, Kumamoto City, HonjoKumamoto, Japan

**Keywords:** Thiamylal, Refractory status epilepticus, Therapeutic drug monitoring, Cytochrome P450, Carbamazepine

## Abstract

**Background:**

Treating refractory status epilepticus (RSE) remains a challenge. Thiamylal can be used as a second- or third-line treatment; however, its potential to induce cytochrome P450 (CYP) activity may reduce the concentration of antiepileptic drugs (AEDs) administered prior to thiamylal. This report details a case of RSE patient treated with thiamylal, with monitored concentrations of thiamylal and other AEDs.

**Case presentation:**

A 72-year-old healthy man developed RSE. Despite the administration of various AEDs, his seizures were not resolved. Thiamylal was then administered at an initial bolus dose of 2.1 mg/kg, followed by a continuous infusion of 4.2–5.2 mg/kg/h. The initial thiamylal concentration was observed at 7.8 μg/mL, increasing to 35.2 μg/mL before decreasing after dose reduction and cessation. Concurrently, the concentration of concomitant carbamazepine decreased from 5.59 μg/mL to 2.1 μg/mL and recovered as thiamylal concentration decreased. Lesser impacts were noted for other AEDs.

**Conclusions:**

This case report underscored the efficacy of thiamylal in treating RSE. However, it also highlighted the need for clinicians to closely monitor the concentrations of concurrent AEDs, especially carbamazepine, during thiamylal therapy.

**Supplementary Information:**

The online version contains supplementary material available at 10.1186/s40780-024-00362-w.

## Background

Refractory status epilepticus (RSE) results from the continuation of status epilepticus (SE) despite the administration of two antiepileptic drugs [1]. Early SE, lasting over 5 min, is reported to develop in 36–80 individuals per 100,000 population, with 6.5–7.2 per 100,000 progressing to RSE [2]. Given that RSE is a life-threatening neurological emergency with a reported fatality rate of 17.65% [1], strategies for its suppression are crucial. While phenobarbital, a classic antiepileptic drug (AED), has resolved 5.3% of RSE cases, newer AEDs including levetiracetam have resolved 41.2% of cases when used after phenytoin as the second-line AED [1]. A randomized controlled trial demonstrated equal probabilities of seizure cessation with levetiracetam (47%), fosphenytoin (45%), and valproate (46%) in patients pre-treated with benzodiazepines [3]. Therefore, despite the advancements in medication for RSE, the need for alternative therapies remains high.

Thiamylal, a type of barbiturate, has been proposed for RSE treatment [4], showing high efficacy as 25 out of 26 pediatric patients were treated successfully over 350 min, although vasopressors and mechanical ventilation were necessary [5]. The studies exhibiting the clinical benefit in adult patients are also available, though relatively sparse [6–8]. Thus, thiamylal may be an effective suppressor of RSE. However, its potential to induce cytochrome P450 (CYP) activity [9] may decrease the concentration and efficacy of concomitant antiepileptics used against RSE. Currently, little information is available regarding the influence of thiamylal on drugs metabolized by CYP.

This case report reviews the potential of thiamylal as a treatment option for RSE, focusing on the measurement of concentrations of thiamylal and two concomitant AEDs in an adult patient with RSE.

## Case presentation

**Fig. 1:**
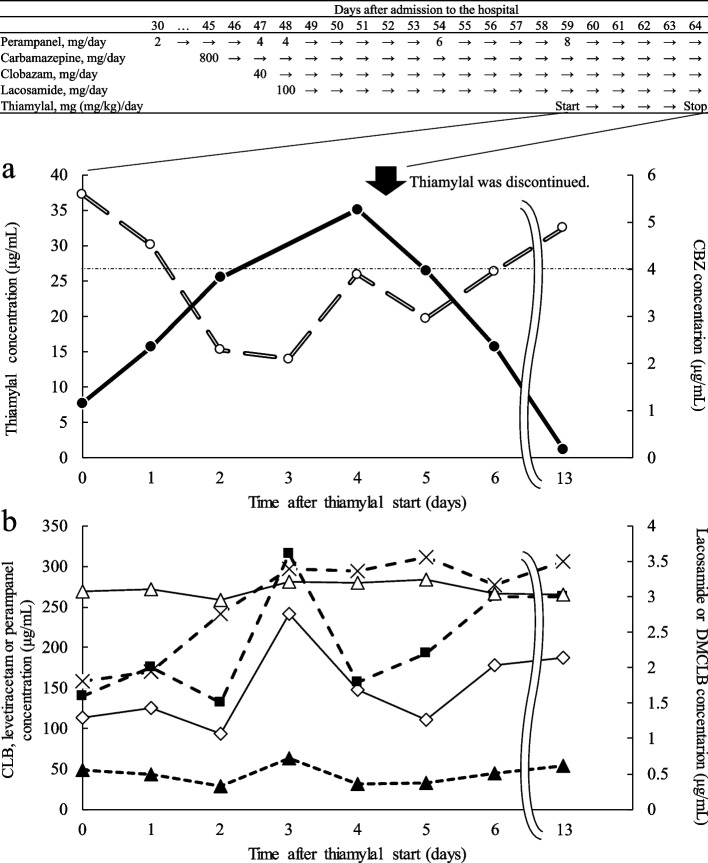
Concentration–time plot for antiepileptic drugs. **a **thiamylal (black circle symbol) and carbamazepine (CBZ, white circle symbol). The horizontal dashed line at the level of 4 μg/mL of carbamazepine depicts the lower limit of effective concentration. **b** clobazam (white diamond symbol) desmethylclobazam (white triangle symbol), perampanel (cross symbol), lacosamide (black square symbol), and levetiracetam (black triangle symbol). Thiamylal was administered as a 150 mg bolus (2.1 mg/kg), followed by 300 mg (4.2 mg/kg) over 90 min, and then continuously at a rate of 375 mg/h (5.2 mg/kg/h)

A 72-year-old healthy man (height: 170 cm, weight: 71.4 kg) was admitted to our hospital due to a relatively rapid deterioration in cognition over four months. Initially treated for meningoencephalitis, the patient presented with convulsions on day 24 and progressed to RSE, necessitating admission to the intensive care unit (ICU). Treatment with methylprednisolone for meningoencephalitis and levetiracetam for convulsions was initiated. Sequentially, the following medications were started as described in Fig. 1 (upper panel): perampanel (2 mg once daily on day 30, increased to 4 mg once daily on day 47, then to 6 mg once daily on day 54, and finally to 8 mg once daily on day 59), carbamazepine (400 mg twice daily, started on day 45), clobazam (20 mg twice daily, started on day 47), and lacosamide (50 mg twice daily, started on day 48). The patient was initially discharged from the ICU on day 51 but returned after 8 days due to a relapse. Thiamylal treatment commenced on day 59, with a loading dose of 150 mg (2.1 mg/kg) as a bolus, followed by a continuous infusion at a rate of 300 mg/h (4.2 mg/kg/h) for 90 min, which was then increased to 375 mg/h (5.2 mg/kg/h).

The serum thiamylal concentration was measured using our validated method, which employs high-performance liquid chromatography with ultraviolet detection at 254 nm (Supplementary Text S1) by referring a report [11]. The initial observation was 7.8 μg/mL in the middle of infusion, immediately before the dose increase to 375 mg/kg/h. It rose to 35.2 μg/mL during the continuous infusion phase and decreased after reducing the dose and cessation on day 63 (Fig. 1a). Concurrently, the concomitant carbamazepine concentration at trough, monitored for clinical decision-making, was found to decrease during the thiamylal infusion from 5.59 μg/mL to 2.1 μg/mL, and recovered as the thiamylal concentration decreased (Fig. 1a). Concentrations of other AEDs—perampanel, clobazam, desmethylclobazam (clobazam's active metabolite), lacosamide, and levetiracetam—were also measured using residual serum samples collected for carbamazepine monitoring. However, lesser impacts on these drugs were observed (Fig. 1b).

From day 58 to day 64, the laboratory test values for liver and kidney function remained stable, with serum albumin ranging from 1.7 to 1.6 g/dL, total bilirubin from 0.1 to 0.4 mg/dL, aspartate transaminase from 16 to 13 U/L, alanine transaminase from 17 to 16 U/L, and the estimated glomerular filtration rate consistently over 90 mL/min/1.73m^2^.

## Discussion and conclusions

This report is the first to observe the concentrations of antiepileptics following the initiation of thiamylal therapy. Notably, carbamazepine levels decreased below the lower limit of effective concentration [10], likely due to thiamylal's induction of CYP enzymes [9]. The concentration of carbamazepine before the start of thiamylal had not been measured. Intriguingly, the impact on carbamazepine appeared to correlate with the concentration of thiamylal, suggesting that thiamylal's CYP induction might be potent at clinical doses. Carbamazepine is primarily metabolized by CYP3A4 and 2C8 into its epoxide form [12], a process that could be enhanced by thiamylal-induced CYP activation. The induction of CYPs generally requires more than several days to complete, while it can already be observed by the second day when inducers such as barbiturates are used. This is supported by in vitro experiments showing that CYP microsomal protein levels doubled by the second day [9]. We observed the elevated blood concentration of carbamazepine at day 4 (Fig. 1). However, we could not identify any incidents that explain the elevation, such as liver damage, concurrent medications that could elevate the levels, and dose escalation of carbamazepine. Concurrent medications were used at stable doses of prednisolone at 35 mg once daily, esomeprazole at 20 mg once daily, alfacalcidol at 1 μg once daily, sulfamethoxazole-trimethoprim at one tablet once daily, carbocysteine at 500 mg thrice daily, mosapride at 5 mg thrice daily, magnesium oxide at 330 mg twice daily, noradrenaline 0.024 μg/kg/min, and midazolam at 0.23 mg/kg/h. Therefore, we have inevitably discussed that the elevation could be attributed to errors such as sampling, dosing, measurement, or intraindividual variability.

However, the influence of thiamylal on other antiepileptics was less pronounced than on carbamazepine. The concentration of perampanel increased at the time of thiamylal administration, which might be largely due to the dose escalation from 6 to 8 mg daily on day 59, coinciding with the initiation of thiamylal, as shown in Fig. 1. An in vitro study demonstrated an increase in messenger RNA levels by 205% for CYP3A4 and 735% for CYP2C19 [13], the main enzymes involved in the metabolism of clobazam and lacosamide. This study also noted 610% increase in CYP2C8, which might account for the differential impact observed. Perampanel, primarily metabolized by CYP3A4 [14], appeared less affected by thiamylal, as its induction impact is smaller compared to CYP2C8 and 2C19. Levetiracetam maintained relatively stable concentrations, attributable to its property of being primarily eliminated by the kidneys, independent of metabolic processes. The sudden increase in concentrations observed in Fig. 1b might be due to the fact that the serum samples were taken specifically for carbamazepine measurement, rather than for general analysis. The relationship between the timing of administration and sampling was not clearly documented in the medical record.

Regarding thiamylal concentration, there is no established therapeutic range for RSE treatment. For brain activity suppression, a concentration of 4.4 μg/mL was identified as the lower limit for the emergence of a burst suppression pattern [15], a threshold reached early in this treatment (Fig. 1a). Ishida et al. reported a median cumulative dose of 27.5 mg/kg over 109.5 min was required to suppress RSE [5], yet in our case, effective treatment was achieved with a lower dose.

In conclusions, this case report highlighted thiamylal's effectiveness in treating RSE. However, clinicians should closely monitor the concentrations of concurrent antiepileptics, particularly carbamazepine, as they may decrease significantly from the start of thiamylal therapy and continue to do so for a few days after its cessation.

### Supplementary Information

Supplementary Material 1.

## Data Availability

Data and materials related to this study are available from the corresponding author upon reasonable request.

## Abbreviations

AED: Antiepileptic drug
*CYP*: Cytochrome P450
ICU: Intensive care unit
RSE: Refractory status epilepticus
SE: Status epilepticus
