# Supplementary material for: Thiamylal serum concentration for refractory convulsive status epilepticus while associated decreased concentrations of concomitant antiepileptics: a case report
Source: J Pharm Health Care Sci. 2024 Jul 12;10:39. doi: 10.1186/s40780-024-00362-w (PMC11245824; doi:10.1186/s40780-024-00362-w)
Supplement: Supplementary file 1 — Supplementary Material 1. [file 40780_2024_362_MOESM1_ESM.docx]

**Online ELECTRONIC supplementary material**

***Journal of Pharmaceutical Health Care and Sciences***

**Thiamylal serum concentration for refractory convulsive status epilepticus while associated decreased concentrations of concomitant antiepileptics: a case report.**

Kazutaka Oda^*^, Tomomi Katanoda, Hitomi Arakaki, Taiki Katsume, Kaho Matsuyama, Hirofumi Jono, and Hideyuki Saito

^*^Corresponding author:

Kazutaka Oda;

E-mail address: kazutakaoda@kuh.kumamoto-u.ac.jp;

Tel: +81-96-373-7457.

**Supplementary Text S1. Measurement method for thiamylal**.

We developed a swift assay method for measuring serum thiamylal concentration using high-performance liquid chromatography (HPLC) equipped with an ultraviolet detection system, supplied by Shimadzu (Kyoto, Japan). The system was operated using LabSolutions version 5.4.2 (analysis GUI) and CBM-20A (management device), controlling the LC-20AD (pump), SIL-20ACHT (auto-injector), SPD-20A (ultraviolet detector), and CTO-10ACvp (column oven). The separation was achieved using a Kinetex EVO C18 column (100 mm × 3.0 mm, 2.6 μm) along with a SecurityGuard Ultra C18 guard column (2.1 mm internal diameter), both acquired from Phenomenex (Torrance, USA).

For the assay, thiamylal, its internal standard (pentobarbital), and human serum were sourced from FUJIFILM Wako Pure Chemical Corporation (Osaka, Japan). We spiked 50 μL serum samples with 5 μL of pentobarbital at a concentration of 20 μg/mL. These samples underwent pretreatment with an equivalent volume of a precipitator for tacrolimus (Alinity i Tacrolimus Whole Blood Precipitation Reagent, from Abbott Japan LLC, Tokyo), followed by 10 seconds of vortexing and centrifugation at 13,000×g for 4 minutes. Subsequently, 5 μL of the supernatant was injected into the system.

The mobile phase comprised a 20 mM sodium-phosphate buffer (pH 2.8) and acetonitrile, starting with a 20% acetonitrile ratio for the first 4 minutes, then increasing to 35% over the next 6 minutes, and finally returning to 20% for the last 5 minutes. We set the column oven temperature at 50°C, the mobile phase flow rate at 0.8 mL/min, and the detection wavelengths at 300 nm for thiamylal and 214 nm for pentobarbital. The calibration range spanned from 0.25 to 32 μg/mL, confirming linearity. Retention and one-cycle times were 9.5 and 4.2 minutes, respectively.

Validation results indicated that the intra-day and inter-day variabilities at concentrations of 0.5, 4, 16, and 32 μg/mL were the ranges of 92.8–102.0%, 97.0–108.5%, and 99.7–107.7%, respectively.
